# Association of Patient-Level and Hospital-Level Factors With Timely Fracture Care by Race

**DOI:** 10.1001/jamanetworkopen.2022.44357

**Published:** 2022-11-30

**Authors:** Ida Leah Gitajn, Paul Werth, Eseosa Fernandes, Sheila Sprague, Nathan N. O'Hara, Sofia Bzovsky, Lucas S. Marchand, Joseph Thomas Patterson, Christopher Lee, Gerard P. Slobogean

**Affiliations:** 1 Dartmouth-Hitchcock Medical Center, Lebanon, New Hampshire; 2 University of Maryland School of Medicine, Baltimore; 3 McMaster University, Hamilton, Ontario, Canada; 4 University of Utah, Salt Lake City; 5 Keck School of Medicine of the University of Southern California, Los Angeles; 6 University of California, Los Angeles

## Abstract

**Importance:**

Racial disparities in treatment benchmarks have been documented among older patients with hip fractures. However, these studies were limited to patient-level evaluations.

**Objective:**

To assess whether disparities in meeting fracture care time-to-surgery benchmarks exist at the patient level or at the hospital or institutional level using high-quality multicenter prospectively collected data; the study hypothesis was that disparities at the hospital-level reflecting structural health systems issues would be detected.

**Design, Setting, and Participants:**

This cohort study was a secondary analysis of prospectively collected data in the PREP-IT (Program of Randomized trials to Evaluate Preoperative antiseptic skin solutions in orthopaedic Trauma) program from 23 sites throughout North America. The PREP-IT trials enrolled patients from 2018 to 2021, and patients were followed for 1-year. All patients with hip and femur fractures enrolled in the PREP-IT program were included in analysis. Data were analyzed April to September 2022.

**Exposures:**

Patient-level and hospital-level race, ethnicity, and insurance status.

**Main Outcomes and Measures:**

Primary outcome measure was time to surgery based on 24-hour time-to-surgery benchmarks. Multilevel multivariate regression models were used to evaluate the association of race, ethnicity, and insurance status with time to surgery. The reported odds ratios (ORs) were per 10% change in insurance coverage or racial composition at the hospital level.

**Results:**

A total of 2565 patients with a mean (SD) age of 64.5 (20.4) years (1129 [44.0%] men; mean [SD] body mass index, 27.3 [14.9]; 83 [3.2%] Asian, 343 [13.4%] Black, 2112 [82.3%] White, 28 [1.1%] other) were included in analysis. Of these patients, 834 (32.5%) were employed and 2367 (92.2%) had insurance; 1015 (39.6%) had sustained a femur fracture, with a mean (SD) injury severity score of 10.4 (5.8). Five hundred ninety-six patients (23.2%) did not meet the 24-hour time-to-operating-room benchmark. After controlling for patient-level characteristics, there was an independent association between missing the 24-hour benchmark and hospital population insurance coverage (OR, 0.94; 95% CI, 0.89-0.98; *P* = .005) and the interaction term between hospital population insurance coverage and racial composition (OR, 1.03; 95% CI, 1.01-1.05; *P* = .03). There was no association between patient race and delay beyond 24-hour benchmarks (OR, 0.96; 95% CI, 0.72-1.29; *P* = .79).

**Conclusions and Relevance:**

In this cohort study, patients who sought care from an institution with a greater proportion of patients with racial or ethnic minority status or who were uninsured were more likely to experience delays greater than the 24-hour benchmarks regardless of the individual patient race; institutions that treat a less diverse patient population appeared to be more resilient to the mix of insurance status in their patient population and were more likely to meet time-to-surgery benchmarks, regardless of patient insurance status or population-based insurance mix. While it is unsurprising that increased delays were associated with underfunded institutions, the association between institutional-level racial disparity and surgical delays implies structural health systems bias.

## Key Points

**Question:**

Are there patient-level or systemic racial disparities in meeting fracture care time-to-surgery benchmarks?

**Findings:**

This cohort study was a secondary analysis of prospectively collected multicenter data for 2565 patients with hip and femur fractures enrolled in 2 randomized trials in the US and Canada. After controlling for patient-level characteristics, there was an association between missing the 24-hour benchmark for surgery and the proportion of insured patients cared for by the hospital, and a separate association between proportion of insured patients and the racial and ethnic minority patients cared for by hospitals.

**Meaning:**

These results suggest that patients seeking care from institutions with more diverse and more uninsured patients are more likely to experience delays greater than the 24-hour benchmarks, regardless of the individual patient race, implying structural health systems biases.

## Teaser

This cohort study examines the association of race, ethnicity, and insurance coverage status at the individual and hospital level with time to surgery among patients with hip and femur fractures.

## Introduction

Racial inequalities relating to health care utilization and outcomes are well documented across many medical specialties,^1,2,3,4^ including orthopedic trauma.^5,6,7,8,9,10,11^ To date, studies in fracture populations have been limited to geriatric patients with hip fractures and have demonstrated that Black patients have a higher likelihood of nonoperative treatment.^12^ Additionally, when Black patients are treated surgically, they experience longer delays to surgery,^6,7,8^ as well as worse postoperative outcomes, including higher rates of revision surgery, readmission, and 1-year mortality.^5^ However, previous studies evaluating racial disparities in trauma have been limited to retrospectively collected data sets at a single site or administrative data sets^5,6,7,12^ that are susceptible to inconsistent reporting and coding inaccuracies.^13,14^

The purpose of the present study was to evaluate a broader patient population undergoing time-sensitive procedures using a prospectively collected data set. To this end, the aims of this study were to evaluate whether (1) there are racial disparities in meeting time-to-operating room benchmarks for time-sensitive hip and femur fractures using high-quality prospectively collected data and (2) delays were attributable to patient factors (ie, race and ethnicity) or institutional factors (ie, racial composition of the patients cared for at an institutional level). A 24-hour time-to-operating room benchmark is well-accepted for both femoral shaft and hip fractures to reduce morbidity and mortality.^7,15,16,17,18,19,20,21,22,23,24,25^ Our hypothesis was that inequities are present, not at the patient level, but at the institutional level in association with underresourced hospitals caring for more diverse populations.

## Methods

### Study Design and Procedures

This cohort study was a secondary analysis of data collected in the Program of Randomized Trials to Evaluate Preoperative Antiseptic Skin Solutions in Orthopaedic Trauma (PREP-IT) program. The PREP-IT trials were approved by a central institutional review board (Advarra). The PREP-IT program is comprised of 2 parallel cluster randomized crossover trials: Aqueous-PREP (Preoperative Aqueous Antiseptic Skin Solutions in Open Fractures) (NCT03385304) and PREPARE (Preoperative Alcohol Skin Solutions in Fractured Extremities) (NCT03523962).^26^ Enrollment took place in 23 sites across the US and Canada. Of these 23 sites, 1 was a military medical center and all others were private nonprofit institutions. Twenty-one sites were designated American College of Surgeons Level I trauma centers and 2 sites were Level II trauma centers. Twenty states or provinces in the US and Canada were represented geographically, with multiple (ie, more than 2) sites in all US Census regions (northeast, south, midwest, west) and in Canada in both urban and rural locations. The PREP-IT trials enrolled patients from 2018 to 2021 and patients were followed for 1 year. PREP-IT trial participants were aged 18 years or older and presented with either an open upper or lower extremity fracture or a closed fracture of the lower extremity or pelvis. This study followed the Strengthening the Reporting of Observational Studies in Epidemiology (STROBE) reporting guideline for cohort studies.

The PREP-IT program compares iodophor-based vs chlorhexidine-based antiseptic skin preparation solutions. Clinical sites were randomized to 1 skin preparation solution and crossed over to the alternative study solution every 2 months. In this cluster randomized crossover trial design, all patients treated at recruiting hospitals received the predetermined study intervention prior to patient enrollment. As a result, consent did not need to occur prior to the patient’s urgent surgery, and study participation had no effect on time to surgery.

For the current cohort study, all patients included in the PREP-IT program who sustained closed femoral shaft (Orthopaedic Trauma Association classification 32A, 32B, 32C, 33A, 33B, or 33C) or closed hip fracture (Orthopaedic Trauma Association classification 31A, 31B, or 31C) were eligible for inclusion. The outcome investigated was time to surgery bifurcated into surgery within 24 hours of hospital admission or greater than 24 hours from hospital admission. No enrolled patients were excluded from the analysis.

Patients were dichotomized as White or racial and ethnic minority patients based on self-reported identification within 12 initial race and ethnic categories. Patients not identifying as White were grouped together in order to cluster all underrepresented minorities who may experience disparities relative to their White counterparts, which resulted in a more straightforward regression model that optimized statistical power and thus ability to draw conclusions. In particular, many of the 12 racial and ethnic categories had quite small patient numbers, which would make comparisons impossible. Hospital population racial composition was defined based on hospital racial and ethnic minority patient population. Insurance status was coded as either insured or uninsured. Both hospital population racial composition and hospital population insurance were calculated as the proportion of patients who identified as a racial and ethnic minority and without insurance, respectively. As an example, a hospital site that treated 10 patients without insurance and 90 patients with insurance would result in 10% of the site’s population (ie, 10/[10 + 90]) being uninsured.

### Statistical Analysis

Univariate tests based on meeting the 24-hour surgical window outcome included independent-sample *t* tests for continuous variables and χ^2^ tests for categorical variables. Considering the nested structure of the data focusing on person-level and institution-level independent variables of the person-level dependent variable, a mixed effects modeling approach was employed for the primary analysis. Toward developing the mixed effects model, we used a model building approach that included stepwise variable block inclusion, largely following the approach outlined, by first modeling steps offered with patient-level variables included and then by introducing institution-level variables (ie, introducing additional complexity at each step).^32^ Specifically, person-level continuous independent variables were mean-centered. Institution-level independent variables were constructed by determining proportion of patients that met the specific criteria (ie, uninsured status and of minority racial or ethnic status) as compared with total number of patients served by each institution. An interaction term was included between institution-level patient makeup by racial and ethnic minority status and insurance status to understand the combined effect on the focal outcome of a patient’s probability of meeting the 24-hour surgical window. Due to the interaction term being significant (*P* < .05 in 2-sided tests), it was further probed via a model-implied plot. Additionally, curvilinear treatment of institution-level racial composition of patients served was empirically tested via the inclusion of quadratic and cubic terms, which did not demonstrate significance either comparing omnibus model fit statistics with the more parsimonious model or individual model parameters. The variance inflation factor was evaluated for all model predictors and met acceptable thresholds (below 5) (eFigure 1 in Supplement 1). Average marginal effects were also derived from the mixed effects model (eTable in Supplement 1).

## Results

A total of 2565 patients from 23 medical centers across the US and Canada were included with a mean (SD) age 64.5 (20.4) years (1129 [44.0%] men; mean [SD] body mass index [BMI; calculated as weight in kilograms divided by height in meters squared], 27.3 [14.9]); 2112 patients (82.3%) were White (the racial and ethnic minority group included 83 patients [3.2%] identifying as Asian, 343 [13.4%] as Black, and 28 [1.1%] as other) (Table 1). Of these patients, 834 (32.5%) were employed and 2367 (92.2%) had insurance; 1015 (39.6%) had sustained a femur fracture, with a mean (SD) injury severity score of 10.4 (5.8).

**Table 1.** Patient Characteristics Stratified by Meeting 24-Hour Time-to-Surgery Benchmarks

Characteristics	Patients, No. (%)			*P* value	SMD
	Total (N = 2566)	Met 24-h benchmark (n = 1970)	Did not meet 24-h benchmark (n = 596)		
Age, mean (SD), y	64.54 (20.44)	62.6 (21.3)	70.9 (17.6)	<.001	0.424
Sex					
Men	1129 (44.0)	890 (45.2)	239 (40.1)	.03	0.103
Women	1437 (56.0)	1080 (54.8)	357 (59.9)		
BMI, mean (SD)	27.26 (14.90)	27.26 (7.25)	27.27 (7.56)	.99	<0.001
BMI category					
Underweight (<18.5)	122 (4.8)	87 (4.4)	35 (5.9)	.52	0.095
Normal weight (18.5-24.9)	987 (38.5)	763 (38.8)	224 (37.6)		
Overweight (25.0-29.9)	749 (29.2)	582 (29.6)	167 (28.0)		
Obesity class					
I (30.0-34.9)	386 (15.0)	291 (14.8)	95 (15.9)		
II (35.0-39.9)	168 (6.5)	133 (6.8)	35 (5.9)		
III (≥40.0)	153 (6.0)	113 (5.7)	40 (6.7)		
Employed	834 (32.5)	710 (36.1)	124 (20.8)	<.001	0.343
Race					
Racial and ethnic minority				.001	0.207
Asian	83 (3.2)	50 (2.5)	33 (5.5)		
Black	343 (13.4)	279 (14.2)	64 (10.7)		
Other^a^	28 (1.1)	22 (1.1)	6 (1.0)		
White	2112 (82.3)	1619 (82.2)	493 (82.7)		
Insurance, yes (%)	2367 (92.2)	1810 (91.9)	557 (93.6)	.21	0.065
ASA class					
I	107 (4.2)	99 (5.0)	8 (1.3)	<.001	0.36
II	803 (31.3)	657 (33.4)	146 (24.5)		
III	1354 (52.8)	1021 (51.8)	333 (55.9)		
IV	297 (11.6)	189 (9.6)	108 (18.1)		
V	5 (0.2)	4 (0.2)	1 (0.2)		
Femur fracture^b^	1015 (39.6)	802 (40.7)	213 (35.7)	.03	0.102
ISS, mean (SD)	10.36 (5.76)	10.31 (5.91)	10.53 (5.25)	.42	0.041
**Hospital-level**					
Patients without insurance, mean (SD), %	7 (7)	8 (7)	6 (6)	<.001	0.333
Racial and ethnic minority patients, mean (SD), %	17 (15)	17 (15)	18 (14)	.23	0.057

Abbreviations: ASA, American Society of Anesthesiologists; BMI, body mass index (calculated as weight in kilograms divided by height in meters squared); ISS, injury severity score; SMD, standardized mean difference.

^a^ Other includes Middle Eastern, Native or indigenous, Central or South American, Latin American, and multiracial.

^b^ Vs hip fractures.

A total of 596 patients (23.2%) did not meet 24-hour time to operating room benchmarks. Patients who did not meet the 24-hour standard-of-care surgical window were frequently older (mean [SD] age, 70.9 [17.6] vs 62.6 [21.3] years; *P* < .001), more likely to be women (357 [59.9%] vs 1080 [54.8%]; *P* = .03), less likely to be employed (124 [20.8%] vs 710 [36.1%]; *P* < .001), had a higher American Society of Anesthesiologists physical status classification (eg, class IV: 108 [18.1%] vs 189 [9.6%]; *P* < .001) and more often suffered a femur fracture as opposed to hip fracture (213 [35.7%] vs 802 [40.7%]; *P* = .03) (Table 1). Distribution of hip and femur fracture is reported in Table 2.

**Table 2.** Frequency Procedures Met 24-Hour Time-to-Surgery Benchmarks by Site

Site	Procedures, No. (%)			Patients, No. (%)	
	Total	Met 24-h benchmark	Did not meet 24-h benchmark	Racial/ethnic minority group	Uninsured
1	83	74 (89.2)	9 (10.8)	6 (7.2)	2 (2.4)
2	86	60 (69.8)	26 (30.2)	7 (8.1)	3 (3.5)
3	99	88 (88.9)	11 (11.1)	0	9 (9.1)
4	92	77 (83.7)	15 (16.3)	26 (28.3)	17 (18.5)
5	76	59 (77.6)	17 (22.4)	8 (10.5)	4 (5.3)
6	64	54 (84.4)	10 (15.6)	8 (12.5)	0
7	176	138 (78.4)	38 (21.6)	17 (9.7)	13 (7.4)
8	77	69 (89.6)	8 (10.4)	8 (10.4)	3 (3.9)
9	80	56 (70.0)	24 (30.0)	1 (1.3)	3 (3.8)
10	152	134 (88.2)	18 (11.8)	28 (18.4)	13 (8.6)
11	116	86 (74.1)	30 (25.9)	61 (52.6)	27 (23.3)
12	71	54 (76.1)	17 (23.9)	30 (42.3)	2 (2.8)
13	91	72 (79.1)	19 (20.9)	53 (58.2)	4 (4.4)
14	235	197 (83.8)	38 (16.2)	65 (27.7)	14 (6.0)
15	433	196 (45.3)	237 (54.7)	65 (15.0)	12 (2.8)
16	38	37 (97.4)	1 (2.6)	7 (18.4)	13 (34.2)
17	31	24 (77.4)	7 (22.6)	7 (22.6)	2 (6.5)
18	169	158 (93.5)	11 (6.5)	2 (1.2)	9 (5.3)
19	82	65 (79.3)	17 (20.7)	29 (35.4)	13 (15.9)
20	86	65 (75.6)	21 (24.4)	0	3 (3.5)
21	107	100 (93.5)	7 (6.5)	4 (3.7)	12 (11.2)
22	4	3 (75.0)	1 (25.0)	2 (50.0)	0
23	118	104 (88.1)	14 (11.9)	20 (17.0)	19 (16.1)

There was variability across sites with regards to meeting 24-hour benchmarks and race and ethnicity distribution, as well as population-based health insurance. Institutions met 24-hour time-to-operating-room benchmarks through a range of 45.2% (196 of 433 procedures) to 97.4% (37 of 38 procedures) (Table 2; eFigure 2 in Supplement 1). Minority race and ethnicity distribution varied from 0% (in 99 procedures) to 58.2% (53 of 91 procedures) of population enrolled in the included institutions (Table 2). Uninsured patients varied from 0% (in 64 procedures) to 34.2% (13 of 38 procedures) of population enrolled in the study at included institutions (Table 2).

After controlling for patient-level characteristics, there was not an independent association between missing 24-hour time-to-surgery benchmark and patient-level insurance status (OR, 0.69; 95% CI, 0.45-1.05; *P* = .08). However, increasing hospital population insurance coverage was protective against missing time-to-surgery benchmarks (OR, 0.94; 95% CI, 0.89-0.98). At the patient level, there was no association between missing 24-hour time-to-surgery benchmark and race or ethnicity (OR, 0.96; 95% CI, 0.72-1.28; *P* = .79) and there was no independent association between hospital population racial composition and surgical delay (OR, 0.97; 95% CI, 0.75-1.24; *P* = .78) (Table 3). There was an independent association between the hospital population insurance coverage and hospital population racial composition as an interaction term, suggesting that there was a moderating effect (OR, 1.03; 95% CI, 1.01-1.05; *P* = .03) (Table 3). These results suggest that patients seeking care from a hospital treating a patient population with a more diverse racial composition and more uninsured patients were at higher risk for missing 24-hour time-to-surgery benchmarks (Figure). Of note, patients seeking care from hospitals treating less diverse patient populations with less insurance coverage were not at increased risk for missing 24-hour time-to-operating room benchmarks (Figure). There was no independent association between risk of missing 24-hour time-to-operating room benchmarks and injury location (hip or femur fracture) (OR, 1.09; 95% CI, 0.86-1.27; *P* = .49). There were independent associations between missing the 24-hour benchmark and age (OR, 1.01; 95% CI, 1.00-1.02; *P* = .003), American Society of Anesthesiologists class, patient employment status (OR, 0.74; 95% CI, 0.56-0.98; *P* = .04), and patient BMI (OR, 1.02; 95% CI, 1.01-1.04, *P* = .04).

**Table 3.** Random Intercepts-Only Binary Logistic Mixed Effects Model Examining Time-to-Surgery

Fixed effects	Received surgery within 24 h, OR (95% CI)	*P* value
Intercept	0.16 (0.04-0.44)	<.001
**Patient characteristics**		
Age^a^	1.01 (1.00-1.02)	.003
Sex		
Women	1 [Reference]	[Reference]
Male	1.04 (0.83-1.29)	.75
Race^b^		
Racial and ethnic minority	1 [Reference]	[Reference]
White	0.96 (0.72-1.29)	.79
Fracture		
Hip	1 [Reference]	[Reference]
Femur	1.09 (0.86-1.37)	.49
ASA class		
I	1 [Reference]	[Reference]
II	2.59 (1.18-5.67)	.02
III	3.11 (1.41-6.84)	.005
IV	5.80 (2.53-13.32)	<.001
Employment		
Not employed	1 [Reference]	[Reference]
Employed	0.74 (0.56-0.98)	.04
Insurance coverage		
Not covered	1 [Reference]	[Reference]
Coverage	0.69 (0.45-1.05)	.08
BMI^a^	1.02 (1.01-1.04)	.006
**Hospital characteristics**		
Population insurance coverage	0.94 (0.89-0.98)	.005
Racial composition	0.97 (0.75-1.24)	.78
Population insurance coverage × diversity	1.03 (1.01-1.05)	.03
**Random effects**		
σ^2^	3.29	NA
τ_00 site_	0.31	NA
ICC	0.09	NA
N _site_	23	NA
Total observations, No.	2558	NA
Marginal *R^2^*/Conditional *R^2^*	0.117/0.192	NA

Abbreviations: BMI, body mass index (calculated as weight in kilograms divided by height in meters squared); ICC, intraclass correlation coefficient; σ^2^, within-hospital variance; NA, not applicable; τ_00_, between-hospital variance.

^a^ Continuous variable centered on hospital site mean.

^b^ Race and ethnicity dichomotomized into White versus racial and ethnic minority categories from 12 initial categories; many of the 12 racial and ethnic categories had quite small patient numbers, which would make comparisons impossible.

**Figure.**
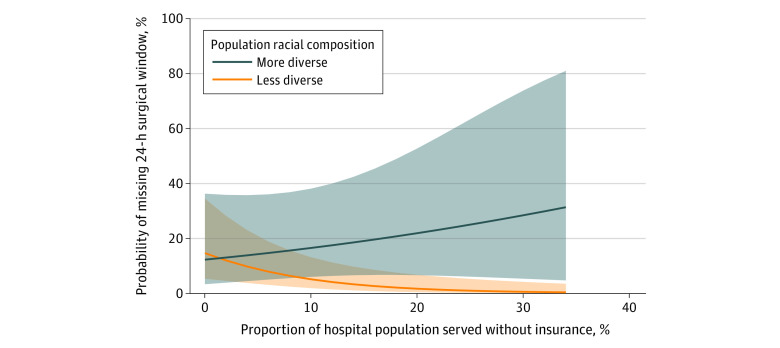
24-Hour Time-to-Surgery Benchmarks for Interaction

At low rates of uninsured patients, the probability of missing the 24-hour time-to-surgery benchmark was 12.5% to 14.6% when racial composition varied from 0% to 50% racial and ethnic minority patients (Figure). Conversely, at higher rates of uninsured patients the risk of missing the 24-hour window was higher among more diverse populations. For example, at 30% uninsured, the risk of missing the 24-hour window was 0.5% (95% CI, 0.1%-34.4%) when racial composition had a low racial and ethnic minority patient population and 17.6% (95% CI, 4.0%-50.3%) at 50% racial and ethnic minority patient composition.

## Discussion

The results of this study demonstrate disparities in meeting fracture care time-to-operating room benchmarks at the hospital level rather than at the patient level. Patients seeking care from hospitals with more diverse and less insured patient populations were at higher risk of delayed surgical treatment beyond 24-hour time-to-operating room benchmarks for both femur and hip fractures. For example, at low rates of uninsured patients, there was minimal difference between probability of missing the 24-hour time-to-surgery benchmark, but at higher rates of uninsured patients, there were stark differences between institutions with different racial composition. At the low end, when racial composition had fewer racial and ethnic minority patients, the probability of missing the 24-hour window was less than 1%; however, as the racial composition of racial and ethnic minority patients increased, the risk of missing the 24-hour window also increased, reaching almost 18% when racial composition was 50% racial and ethnic minority patients. Hospitals that treated less diverse (ie, larger proportions of White) patient populations appeared to be more resilient to the mix of insurance status of their patient population, and did not have an association between worsening delay to surgery with increasing uninsured patient population. It is likely that well-funded health systems caring for a higher proportion of insured patients have in place quality improvement programs and other support structures (such as operating room access) that ensure appropriate time-to-operating room for these time-sensitive procedures, and that these programs may be missing at institutions caring for more racially diverse and less insured patient populations. More research is needed to explore health system programs or structures that explain these findings.

This analysis also demonstrated that increasing age, increasing medical comorbidities (reflected in ASA class), and increasing BMI were, not surprisingly, associated with increased risk of missing the 24-hour time to surgery benchmark, as these patients likely required more extensive medical optimization. Additionally, employed patients were less likely to miss this benchmarks. These results likely reflect the relative health and vigor of the patient cohort (the converse of age and medical comorbidities).

The results of this study are consistent with prior studies evaluating disparities in orthopedic care on a systems or institutional scale as well as those evaluating disparities on a patient-level.^8,27^ Two prior studies have demonstrated racial disparities at the structural health systems level both in time to surgery as well as hospital length of stay for hip fractures.^8,27^ The results of this study are also consistent with several prior studies demonstrating delays in hip fracture treatment among racial and ethnic minority patients^6,8,12,27,28^ and worse outcomes among racial and ethnic minority patients.^5,6,8,9,10,11,28^ As these studies only evaluated disparities on the patient level, it is impossible to know whether their findings were due to patient-specific biases or structural health systems issues. The results of this study were also consistent with a study within the Kaiser Permanente patient population demonstrating that in their integrated managed care system, in which all patients have health insurance coverage, racial and ethnic minority patients with hip fracture had postoperative mortality rates similar to, or lower, than White patients.^30^ In this study, hospitals attending to a population with more insured patients were more effective at meeting time-to-operating room benchmarks regardless of hospital racial composition, which was consistent with the Kaiser Permanente system where all patients are insured.

The results of this study can explain, at least in part, the results of a survey of the American Association of Orthopaedic Surgery, which found that only 9% of orthopedic surgeons believe that race or ethnicity influences orthopedic care that is provided.^29^ It may be that it is more difficult for clinicians to detect disparities that occur as a result of hospital-level systemic issues as opposed to patient-by-patient disparities.

The disparities identified in this, and prior investigations, signal an alarming deficiency of the US health care system. While identification of health disparities is a critical first step, understanding reasons for these disparities is critical to progress toward a more equitable health care system. This and other analyses^8,27^ suggest that racial disparities may be occurring on a health systems level as opposed to a patient-by-patient level. Targeting health care systems for quality improvement and public health initiatives is critical to improving health care equity.

### Strengths and Limitations

There are several strengths associated with this study. This study leveraged a high-quality prospectively collected, adjudicated data set that had advantages in validity over prior research using administrative data (such as the National Inpatient Sample, Trauma Quality Improvement Program, American College of Surgeons National Surgical Quality Improvement Program or New York State’s Statewide Planning and Research Cooperative System)^5,8,12,27,28^ that are subject to issues with inconsistent reporting and coding inaccuracies^13,14^ or single center studies.^6,31^ Furthermore, this analysis included several time-sensitive fractures, both of which demonstrated the same effect in the primary analysis suggesting that this association was more widespread across injuries and not limited to a specific injury type. Additionally, the multicenter nature of this study allowed for evaluation of disparities on a national scale, as opposed to a single center.

There are several limitations associated with this study. The results of this study may not be generalizable to all institutions since the data for this study was derived from a prospective study at private nonprofit Level I and II trauma centers with funded research infrastructure. It likely that institutions with a strong research infrastructure are better resourced than institutions without a strong resource infrastructure, suggesting that these results may be even more profound across a broader range of institutions. We used a single model approach for both hip and femur fractures because we were adjusting for the same set of independent variables. This has the benefit of a larger sample size and thus more statistical power, which helps alleviate issues associated with imbalance in the outcome. Furthermore, fracture location (femur vs hip) was included in the multivariate model and there was no association between fracture location and time-to-operating room. Because the PREPARE trial was not designed to assess racial disparities for time-sensitive procedures, the groups were not evenly distributed and there were differences between patient cohorts who met the 24-hour time-to-surgery benchmark and those that did not. However, multivariate regression was used to control for these differences, as is indicated for observational research.

## Conclusions

This cohort study demonstrated that patients seeking care from an institution with a patient population that was more racially and ethnically diverse and greater proportions of uninsured patients were more likely to experience delays greater than the 24-hour time-to-surgery benchmark, regardless of the individual patient race or ethnicity. Furthermore, institutions that treated a less diverse patient population appeared to be more resilient to the mix of insurance status in their patient population and were able to meet time-to-operating room benchmarks regardless of patient insurance status or population-based insurance mix. While increased delays in association with a poorer payor mix would not be surprising, the fact that delays are only seen in association with a more racially diverse patient population can only be explained by structural health systems issues.

## Notes

Supplement 1. **eTable.** Average Marginal Estimates Follow-up to Mixed Effects Model Examining Time-To-Surgery **eFigure 1.** Variance Inflation Factor for All Model Predictors **eFigure 2.** Odds Ratios for Meeting Time-to-Operating Room Benchmarks per Site

Supplement 2.
Nonauthor Collaborators

Supplement 3.
Data Sharing Statement
