# Supplementary material for: Association of Patient-Level and Hospital-Level Factors With Timely Fracture Care by Race
Source: JAMA Netw Open. 2022 Nov 30;5(11):e2244357. doi: 10.1001/jamanetworkopen.2022.44357 (PMC9713603; doi:10.1001/jamanetworkopen.2022.44357)
Supplement: Supplement 1. — eTable. Average Marginal Estimates Follow-up to Mixed Effects Model Examining Time-To-Surgery eFigure 1. Variance Inflation Factor for All Model Predictors eFigure 2. Odds Ratios for Meeting Time-to-Operating Room Benchmarks per Site [file jamanetwopen-e2244357-s001.pdf]

Supplemental Online Content

Gitajn IL, Werth P, Fernandes E, et al; PREP-IT Investigators. Association of patient-level and hospital-level factors with timely fracture care by race. *JAMA Netw Open.* 2022;5(11):e2244357. doi:10.1001/jamanetworkopen.2022.44357

eTable. Average Marginal Estimates Follow-up to Mixed Effects Model Examining Time-To-Surgery

eFigure 1. Variance Inflation Factor for All Model Predictors

eFigure 2. Odds Ratios for Meeting Time-to-Operating Room Benchmarks per Site

This supplemental material has been provided by the authors to give readers additional information about their work.

© 2022 Gitajn IL et al. *JAMA Network Open.*

**eTable.** *Average Marginal Estimates Follow-up to Mixed Effects Model Examining Time-To-Surgery*			
**Predictor**		**AME**[1]	**95% CI**
Age		0.2%	0.1 - 0.3
Sex: *Male*		0.5%	-2.7 – 3.8
Race: *White*		-0.6%	-5.0 – 3.8
Fracture Type: *Femur*		1.2%	-2.3 – 4.7
ASA: *Class II*		10.5%	3.6 – 17.4
ASA: *Class III*		13.1%	6.2 – 20.1
ASA: *Class IV*		23.8%	15.1 – 32.5
Employed: *Yes*		-4.4%	-8.4 – -0.4
Insurance: *Yes*		-6.0%	-13.1 – 1.1
Body Mass Index		0.3%	0.1 – 0.5
Hospital Population Insurance Coverage		-1.0%	-1.7 – -0.3
Hospital Population Diversity		2.1%	-0.5 – 4.7
**Interaction**	Hospital Population Diversity		
Hospital Population Insurance Coverage	(-)1 SD	-1.4%	-2.4 – -0.5
	Mean	-0.9%	-1.6 – -0.3
	(+)1 SD	-0.3%	-1.1 – 0.4
Note: [1]Average marginal estimates.			

© 2022 Gitajn IL et al. *JAMA Network Open.*

eFigure 1. Variance inflation factor for all model predictors

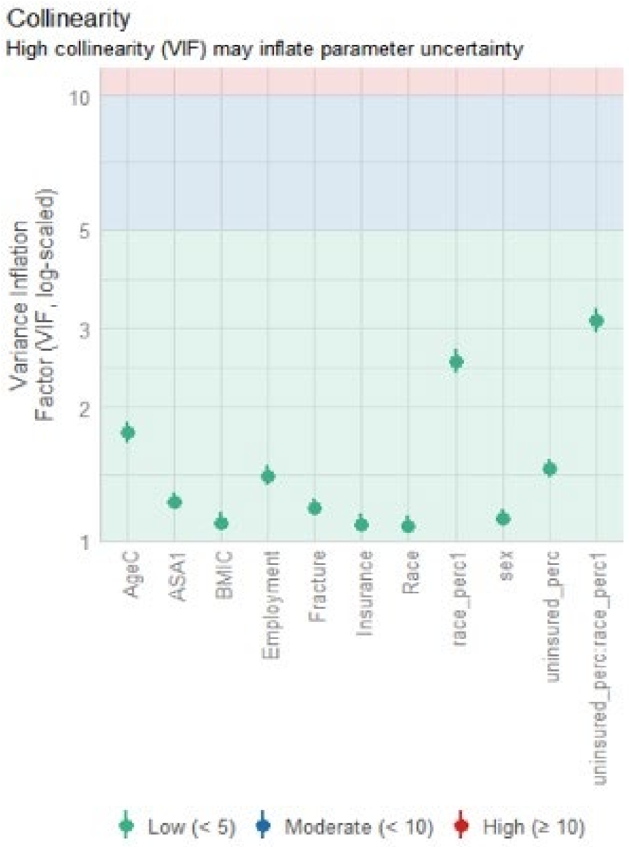

© 2022 Gitajn IL et al. *JAMA Network Open.*

eFigure 2. Odds ratios for meeting time-to-operating room benchmarks per site

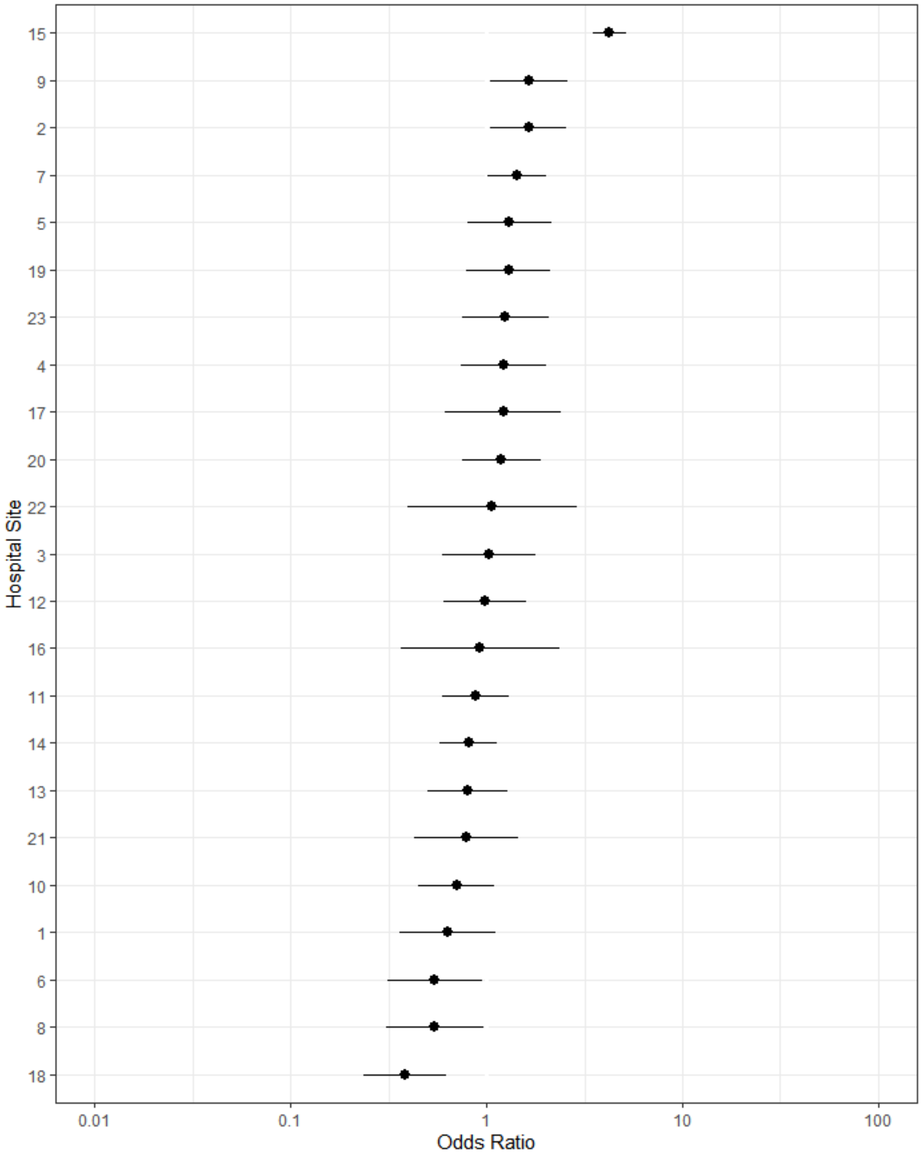

© 2022 Gitajn IL et al. *JAMA Network Open.*
