# Supplementary material for: Association of Patient-Level and Hospital-Level Factors With Timely Fracture Care by Race
Source: JAMA Netw Open. 2022 Nov 30;5(11):e2244357. doi: 10.1001/jamanetworkopen.2022.44357 (PMC9713603; doi:10.1001/jamanetworkopen.2022.44357)
Supplement: Supplement 2. — Nonauthor Collaborators [file jamanetwopen-e2244357-s002.pdf]

Supplemental Online Content: Nonauthor Collaborators

*Indicates required information. Only first name, last name, and suffix will appear in PubMed.

***Group Name(s): PREP-IT Investigators**

***First Name and Middle Initial(s)**	***Last Name**	***Suffix (eg, Jr, III)**	Academic Degrees	Institution	Location (city, state/province, country)	Role or Contribution, eg, chair, principal investigator	Group (if more than 1 Group listed in the byline) and/or Subgroup (eg, Steering Committee)
Gerard P	Slobogean		MD	University of Maryland School of Medicine	Baltimore, Maryland	Principal Investigator	Executive Committee, Steering Committee
Sheila	Sprague		PhD	McMaster University	Hamilton, Ontario	Principal Investigator	Executive Committee, Steering Committee
Jeffrey	Wells			Trauma Survivors Network	Falls Church, Virginia	Patient Representative	Executive Committee, Steering Committee, Patient Centred Outcomes Core
Mohit	Bhandari		MD PhD	McMaster University	Hamilton, Ontario	Principal Investigator	Executive Committee, Steering Committee
Anthony D	Harris			University of Maryland School of Medicine	Baltimore, MD	Committee Member	Steering Committee, Adjudication Committee
C Daniel	Mullins			University of Maryland	Baltimore, MD	Committee Member	Steering Committee
Lehana	Thabane			McMaster University	Hamilton, Ontario	Committee Member	Steering Committee
Amber	Wood			Association of periOperative Registered Nurses	Denver, CO	Committee Member	Steering Committee
Gregory J	Della Rocca			University of Missouri	Columbia, MO	Chair	Adjudication Committee
Joan	Hebden			University of Maryland	Baltimore, MD	Committee Member	Adjudication Committee
Kyle J	Jeray			Greenville Health System	Greenville, SC	Committee Member	Adjudication Committee
Lucas S	Marchand			University of Maryland	Baltimore, MD	Committee Member	Adjudication Committee
Lyndsay M	O'Hara			University of Maryland School of Medicine	Baltimore, MD	Committee Member	Adjudication Committee
Robert	Zura			LSU Health	New Orleans, LA	Committee Member	Adjudication Committee
Christopher	Lee			University of California	Los Angeles, CA	Committee Member	Adjudication Committee
Joseph	Patterson			University of Southern California	Los Angeles, CA	Committee Member	Adjudication Committee
Michael J	Gardner			Stanford University School of Medicine	Palo Alto, CA	Chair	Data and Safety Monitoring Committee
Jenna	Blasman				Kitchener, ON	Patient Representative	Data and Safety Monitoring Committee

Supplemental Online Content: Nonauthor Collaborators

*Indicates required information. Only first name, last name, and suffix will appear in PubMed.

***First Name and Middle Initial(s)**	***Last Name**	***Suffix (eg, Jr, III)**	Academic Degrees	Institution	Location (city, state/province, country)	Role or Contribution, eg, chair, principal investigator	Group (if more than 1 Group listed in the byline) and/or Subgroup (eg, Steering Committee)
Jonah	Davies			University of Washington	Seattle, WA	Committee Member	Data and Safety Monitoring Committee
Stephen	Liang			Washington University	St. Louis, MO	Committee Member	Data and Safety Monitoring Committee
Monica	Taljaard			Ottawa Hospital Research Institute	Ottawa, ON	Committee Member	Data and Safety Monitoring Committee
PJ	Devereaux			McMaster University	Hamilton, Ontario	Committee Member	Research Methodology Core
Gordon H	Guyatt			McMaster University	Hamilton, Ontario	Committee Member	Research Methodology Core
Lehana	Thabane			McMaster University	Hamilton, Ontario	Committee Member	Research Methodology Core
Debra	Marvel				Baltimore, MD	Patient Representative	Patient Centred Outcomes Core
Jana	Palmer				Baltimore, MD	Patient Representative	Patient Centred Outcomes Core
Jeff	Friedrich			Slate Magazine	Washington, DC	Editor	Patient Centred Outcomes Core
C Daniel	Mullins			University of Maryland	Baltimore, MD	Core Member	Patient Centred Outcomes Core
Nathan N	O'Hara			University of Maryland School of Medicine	Baltimore, MD	Core Member	Patient Centred Outcomes Core
Ms Frances	Grissom			Trauma Survivor Network	Baltimore, MD	Core Member	Patient Centred Outcomes Core
Gregory J	Della Rocca			University of Missouri	Columbia, MO	Core Member	Orthopaedic Surgery Core
I Leah	Gitajn			Dartmouth University	Hanover, NH	Core Member	Orthopaedic Surgery Core
Kyle J	Jeray			Greenville Health System	Greenville, SC	Core Member	Orthopaedic Surgery Core

*Indicates required information. Only first name, last name, and suffix will appear in PubMed.

***First Name and Middle Initial(s)**	***Last Name**	***Suffix (eg, Jr, III)**	Academic Degrees	Institution	Location (city, state/province, country)	Role or Contribution, eg, chair, principal investigator	Group (if more than 1 Group listed in the byline) and/or Subgroup (eg, Steering Committee)
Saam	Morshed			San Francisco General Hospital	San Francisco, CA	Core Member	Orthopaedic Surgery Core
Robert V	O'Toole			University of Maryland School of Medicine	Baltimore, MD	Core Member	Orthopaedic Surgery Core
Bradley A	Petrisor			Hamilton Health Sciences	Hamilton, Ontario	Core Member	Orthopaedic Surgery Core
Franca	Mossuto			Hamilton Health Sciences	Hamilton, Ontario	Core Member	Operating Room Core
Anthony D	Harris			University of Maryland School of Medicine	Baltimore, MD	Core Member	Infectious Disease Core
Manjari G	Joshi			University of Maryland School of Medicine	Baltimore, MD	Core Member	Infectious Disease Core
Jean Claude	D'Alleyrand			Walter Reed National Military Medical Center	Bethesda, MD	Core Member	Military Core
Justin	Fowler			United States Army	USA	Core Member	Military Core
Jessica	Rivera			San Antonio Military Medical Center	San Antonio, TX	Core Member	Military Core
Max	Talbot			Canadian Armed Forces	Montreal, QC	Core Member	Military Core
Sheila	Sprague		PhD	McMaster University	Hamilton, Ontario	Principal Investigator	McMaster University Methods Centre
Mohit	Bhandari		MD PhD	McMaster University	Hamilton, Ontario	Principal Investigator	McMaster University Methods Centre
Shannon	Dodds			McMaster University	Hamilton, Ontario	Research Coordinator	McMaster University Methods Centre
Silvia	Li		MSc	McMaster University	Hamilton, Ontario	Research Coordinator	McMaster University Methods Centre
David	Pogorzelski			McMaster University	Hamilton, Ontario	Research Coordinator	McMaster University Methods Centre
Alejandra	Rojas			McMaster University	Hamilton, Ontario	Research Coordinator	McMaster University Methods Centre
Gina	Del Fabbro		BPH	McMaster University	Hamilton, Ontario	Research Coordinator	McMaster University Methods Centre

Supplemental Online Content: Nonauthor Collaborators

*Indicates required information. Only first name, last name, and suffix will appear in PubMed.

***First Name and Middle Initial(s)**	***Last Name**	***Suffix (eg, Jr, III)**	Academic Degrees	Institution	Location (city, state/province, country)	Role or Contribution, eg, chair, principal investigator	Group (if more than 1 Group listed in the byline) and/or Subgroup (eg, Steering Committee)
Olivia P	Szasz			McMaster University	Hamilton, Ontario	Research Coordinator	McMaster University Methods Centre
Diane	Heels Ansdell			McMaster University	Hamilton, Ontario	Statistician	McMaster University Methods Centre
Paula	McKay		BSc	McMaster University	Hamilton, Ontario	Manager	McMaster University Methods Centre
Kevin	Murphy			McMaster University	Hamilton, Ontario	Research Coordinator	McMaster University Methods Centre
Gerard P	Slobogean			University of Maryland School of Medicine	Baltimore, MD	Principal Investigator	University of Maryland School of Medicine Administrative Center
Nathan N	O'Hara			University of Maryland School of Medicine	Baltimore, MD	Manager	University of Maryland School of Medicine Administrative Center
Andrea	Howe			University of Maryland School of Medicine	Baltimore, MD	Project Manager	University of Maryland School of Medicine Administrative Center
Haley	Demyanovich			University of Maryland School of Medicine	Baltimore, MD	Project Manager	University of Maryland School of Medicine Administrative Center
C Daniel	Mullins			Univeristy of Maryland School of Pharmacy	Baltimore, MD	Executive Director	University of Maryland School of Pharmacy, The PATIENTS Program
Eric	Kettering			Univeristy of Maryland School of Pharmacy	Baltimore, MD	Senior Instructional Technology and Dissemination Specialist	University of Maryland School of Pharmacy, The PATIENTS Program
Genevieve	Polk			Univeristy of Maryland School of Pharmacy	Baltimore, MD	Assistant Director, Dissemination and Research	University of Maryland School of Pharmacy, The PATIENTS Program

Supplemental Online Content: Nonauthor Collaborators

*Indicates required information. Only first name, last name, and suffix will appear in PubMed.

*First Name and Middle Initial(s)	*Last Name	*Suffix (eg, Jr, III)	Academic Degrees	Institution	Location (city, state/province, country)	Role or Contribution, eg, chair, principal investigator	Group (if more than 1 Group listed in the byline) and/or Subgroup (eg, Steering Committee)
Michelle	Medeiros			Univeristy of Maryland School of Pharmacy	Baltimore, MD	Director of Research	University of Maryland School of Pharmacy, The PATIENTS Program
**PREP IT Clinical Sites:**							
Robert V	O'Toole			University of Maryland School of Medicine, R Adams Cowley Shock Trauma Center	Baltimore, MD	Co-Investigator	Lead Clinical Site (Aqueous-PREP and PREPARE)
Jean Claude	D'Alleyrand			University of Maryland School of Medicine, R Adams Cowley Shock Trauma Center	Baltimore, MD	Co-Investigator	Lead Clinical Site (Aqueous-PREP and PREPARE)
Andrew	Eglseder			University of Maryland School of Medicine, R Adams Cowley Shock Trauma Center	Baltimore, MD	Co-Investigator	Lead Clinical Site (Aqueous-PREP and PREPARE)
Aaron	Johnson			University of Maryland School of Medicine, R Adams Cowley Shock Trauma Center	Baltimore, MD	Co-Investigator	Lead Clinical Site (Aqueous-PREP and PREPARE)
Christopher	Langhammer			University of Maryland School of Medicine, R Adams Cowley Shock Trauma Center	Baltimore, MD	Co-Investigator	Lead Clinical Site (Aqueous-PREP and PREPARE)
Christopher	Lebrun			University of Maryland School of Medicine, R Adams Cowley Shock Trauma Center	Baltimore, MD	Co-Investigator	Lead Clinical Site (Aqueous-PREP and PREPARE)
Jason	Nascone			University of Maryland School of Medicine, R Adams Cowley Shock Trauma Center	Baltimore, MD	Co-Investigator	Lead Clinical Site (Aqueous-PREP and PREPARE)
Raymond	Pensy			University of Maryland School of Medicine, R Adams Cowley Shock Trauma Center	Baltimore, MD	Co-Investigator	Lead Clinical Site (Aqueous-PREP and PREPARE)

*Indicates required information. Only first name, last name, and suffix will appear in PubMed.

***First Name and Middle Initial(s)**	***Last Name**	***Suffix (eg, Jr, III)**	Academic Degrees	Institution	Location (city, state/province, country)	Role or Contribution, eg, chair, principal investigator	Group (if more than 1 Group listed in the byline) and/or Subgroup (eg, Steering Committee)
Andrew	Pollak			University of Maryland School of Medicine, R Adams Cowley Shock Trauma Center	Baltimore, MD	Co-Investigator	Lead Clinical Site (Aqueous-PREP and PREPARE)
Marcus	Sciadini			University of Maryland School of Medicine, R Adams Cowley Shock Trauma Center	Baltimore, MD	Co-Investigator	Lead Clinical Site (Aqueous-PREP and PREPARE)
Gerard P	Slobogean			University of Maryland School of Medicine, R Adams Cowley Shock Trauma Center	Baltimore, MD	Local Principal Investigator	Lead Clinical Site (Aqueous-PREP and PREPARE)
Yasmin	Degano			University of Maryland School of Medicine, R Adams Cowley Shock Trauma Center	Baltimore, MD	Research Assistant	Lead Clinical Site (Aqueous-PREP and PREPARE)
Haley K	Demyanovich			University of Maryland School of Medicine, R Adams Cowley Shock Trauma Center	Baltimore, MD	Research Coordinator	Lead Clinical Site (Aqueous-PREP and PREPARE)
Andrea	Howe			University of Maryland School of Medicine, R Adams Cowley Shock Trauma Center	Baltimore, MD	Research Coordinator	Lead Clinical Site (Aqueous-PREP and PREPARE)
Nathan N	O'Hara			University of Maryland School of Medicine, R Adams Cowley Shock Trauma Center	Baltimore, MD	Manager	Lead Clinical Site (Aqueous-PREP and PREPARE)
Heather	Phipps			University of Maryland School of Medicine, R Adams Cowley Shock Trauma Center	Baltimore, MD	Research Coordinator	Lead Clinical Site (Aqueous-PREP and PREPARE)
Eric	Hempen			University of Maryland School of Medicine, R Adams Cowley Shock Trauma Center	Baltimore, MD	Research Coordinator	Lead Clinical Site (Aqueous-PREP and PREPARE)
Brad A	Petrisor			Hamilton Health Sciences – General Site	Hamilton, ON	Local Principal Investigator	Aqueous-PREP and PREPARE
Herman	Johal			Hamilton Health Sciences – General Site	Hamilton, ON	Local Principal Investigator	Aqueous-PREP and PREPARE

*Indicates required information. Only first name, last name, and suffix will appear in PubMed.

*First Name and Middle Initial(s)	*Last Name	*Suffix (eg, Jr, III)	Academic Degrees	Institution	Location (city, state/province, country)	Role or Contribution, eg, chair, principal investigator	Group (if more than 1 Group listed in the byline) and/or Subgroup (eg, Steering Committee)
Bill	Ristevski			Hamilton Health Sciences – General Site	Hamilton, ON	Co-Investigator	Aqueous-PREP and PREPARE
Dale	Williams			Hamilton Health Sciences – General Site	Hamilton, ON	Co-Investigator	Aqueous-PREP and PREPARE
Matthew	Denkers			Hamilton Health Sciences – General Site	Hamilton, ON	Co-Investigator	Aqueous-PREP and PREPARE
Krishan	Rajaratnam			Hamilton Health Sciences – General Site	Hamilton, ON	Co-Investigator	Aqueous-PREP and PREPARE
Jamal	Al-Asiri			Hamilton Health Sciences – General Site	Hamilton, ON	Co-Investigator	Aqueous-PREP and PREPARE
Jodi	Gallant			Hamilton Health Sciences – General Site	Hamilton, ON	Clinical Research Coordinator	Aqueous-PREP and PREPARE
Kaitlyn	Pusztai			Hamilton Health Sciences – General Site	Hamilton, ON	Clinical Research Coordinator	Aqueous-PREP and PREPARE
Sarah	MacRae			Hamilton Health Sciences – General Site	Hamilton, ON	Clinical Research Coordinator	Aqueous-PREP and PREPARE
Sara	Renaud			Hamilton Health Sciences – General Site	Hamilton, ON	Clinical Research Coordinator	Aqueous-PREP and PREPARE
Roman N	Natoli			IU Health Methodist Hospital	Indianapolis, IN	Local Principal Investigator	Aqueous-PREP and PREPARE
Todd O	McKinley			IU Health Methodist Hospital	Indianapolis, IN	Co-Investigator	Aqueous-PREP and PREPARE
Walter W	Virkus			IU Health Methodist Hospital	Indianapolis, IN	Co-Investigator	Aqueous-PREP and PREPARE
Anthony T	Sorkin			IU Health Methodist Hospital	Indianapolis, IN	Co-Investigator	Aqueous-PREP and PREPARE
Jan P	Szatkowski			IU Health Methodist Hospital	Indianapolis, IN	Co-Investigator	Aqueous-PREP and PREPARE
Brian H	Mullis			IU Health Methodist Hospital	Indianapolis, IN	Co-Investigator	Aqueous-PREP and PREPARE

*Indicates required information. Only first name, last name, and suffix will appear in PubMed.

*First Name and Middle Initial(s)	*Last Name	*Suffix (eg, Jr, III)	Academic Degrees	Institution	Location (city, state/province, country)	Role or Contribution, eg, chair, principal investigator	Group (if more than 1 Group listed in the byline) and/or Subgroup (eg, Steering Committee)
Yohan	Jang			IU Health Methodist Hospital	Indianapolis, IN	Co-Investigator	Aqueous-PREP and PREPARE
Luke A	Lopas			IU Health Methodist Hospital	Indianapolis, IN	Research Coordinator	Aqueous-PREP and PREPARE
Lauren C	Hill			IU Health Methodist Hospital	Indianapolis, IN	Research Coordinator	Aqueous-PREP and PREPARE
Courteney L	Fentz			IU Health Methodist Hospital	Indianapolis, IN	Research Coordinator	Aqueous-PREP and PREPARE
Maricela M	Diaz			IU Health Methodist Hospital	Indianapolis, IN	Research Coordinator	Aqueous-PREP and PREPARE
Krista	Brown			IU Health Methodist Hospital	Indianapolis, IN	Research Coordinator	Aqueous-PREP and PREPARE
Katelyn M	Garst			IU Health Methodist Hospital	Indianapolis, IN	Research Coordinator	Aqueous-PREP and PREPARE
Emma W	Denari			IU Health Methodist Hospital	Indianapolis, IN	Research Coordinator	Aqueous-PREP and PREPARE
Patrick	Osborn			San Antonio Military Medical Center	San Antonio, TX	Local Principal Investigator	Aqueous-PREP and PREPARE
Justin	Fowler			San Antonio Military Medical Center	San Antonio, TX	Co-Investigator	Aqueous-PREP and PREPARE
Sarah	Pierrie			San Antonio Military Medical Center	San Antonio, TX	Local Principal Investigator/Co-Investigator	Aqueous-PREP and PREPARE
Maria	Herrera			San Antonio Military Medical Center	San Antonio, TX	Research Coordinator	Aqueous-PREP and PREPARE
Kyle J	Jeray			Prisma Health - Upstate	Greenville, SC	Local Principal Investigator	Aqueous-PREP and PREPARE
John D	Adams			Prisma Health - Upstate	Greenville, SC	Co-Investigator	Aqueous-PREP and PREPARE
Michael L	Beckish			Prisma Health - Upstate	Greenville, SC	Co-Investigator	Aqueous-PREP and PREPARE

Supplemental Online Content: Nonauthor Collaborators

*Indicates required information. Only first name, last name, and suffix will appear in PubMed.

***First Name and Middle Initial(s)**	***Last Name**	***Suffix (eg, Jr, III)**	Academic Degrees	Institution	Location (city, state/province, country)	Role or Contribution, eg, chair, principal investigator	Group (if more than 1 Group listed in the byline) and/or Subgroup (eg, Steering Committee)
Christopher C	Bray			Prisma Health - Upstate	Greenville, SC	Co-Investigator	Aqueous-PREP and PREPARE
Timothy R	Brown			Prisma Health - Upstate	Greenville, SC	Co-Investigator	Aqueous-PREP and PREPARE
Andrew W	Cross			Prisma Health - Upstate	Greenville, SC	Co-Investigator	Aqueous-PREP and PREPARE
Timothy	Dew			Prisma Health - Upstate	Greenville, SC	Co-Investigator	Aqueous-PREP and PREPARE
Gregory K	Faucher			Prisma Health - Upstate	Greenville, SC	Co-Investigator	Aqueous-PREP and PREPARE
Richard W	Gurich	Jr		Prisma Health - Upstate	Greenville, SC	Co-Investigator	Aqueous-PREP and PREPARE
David E	Lazarus			Prisma Health - Upstate	Greenville, SC	Co-Investigator	Aqueous-PREP and PREPARE
S John	Millon			Prisma Health - Upstate	Greenville, SC	Co-Investigator	Aqueous-PREP and PREPARE
M Christian	Moody			Prisma Health - Upstate	Greenville, SC	Co-Investigator	Aqueous-PREP and PREPARE
M Jason	Palmer			Prisma Health - Upstate	Greenville, SC	Co-Investigator	Aqueous-PREP and PREPARE
Scott E	Porter			Prisma Health - Upstate	Greenville, SC	Co-Investigator	Aqueous-PREP and PREPARE
Thomas M	Schaller			Prisma Health - Upstate	Greenville, SC	Co-Investigator	Aqueous-PREP and PREPARE
Michael S	Sridhar			Prisma Health - Upstate	Greenville, SC	Co-Investigator	Aqueous-PREP and PREPARE
John L	Sanders			Prisma Health - Upstate	Greenville, SC	Co-Investigator	Aqueous-PREP and PREPARE
L Edwin	Rudisill	Jr		Prisma Health - Upstate	Greenville, SC	Co-Investigator	Aqueous-PREP and PREPARE

Supplemental Online Content: Nonauthor Collaborators

*Indicates required information. Only first name, last name, and suffix will appear in PubMed.

***First Name and Middle Initial(s)**	***Last Name**	***Suffix (eg, Jr, III)**	Academic Degrees	Institution	Location (city, state/province, country)	Role or Contribution, eg, chair, principal investigator	Group (if more than 1 Group listed in the byline) and/or Subgroup (eg, Steering Committee)
Michael J	Garitty			Prisma Health - Upstate	Greenville, SC	Co-Investigator	Aqueous-PREP and PREPARE
Andrew S	Poole			Prisma Health - Upstate	Greenville, SC	Co-Investigator	Aqueous-PREP and PREPARE
Michael L	Sims			Prisma Health - Upstate	Greenville, SC	Co-Investigator	Aqueous-PREP and PREPARE
Clark M	Walker			Prisma Health - Upstate	Greenville, SC	Co-Investigator	Aqueous-PREP and PREPARE
Robert	Carlisle			Prisma Health - Upstate	Greenville, SC	Co-Investigator	Aqueous-PREP and PREPARE
Erin A	Hofer			Prisma Health - Upstate	Greenville, SC	Co-Investigator	Aqueous-PREP and PREPARE
Brandon	Huggins			Prisma Health - Upstate	Greenville, SC	Co-Investigator	Aqueous-PREP and PREPARE
Michael	Hunter			Prisma Health - Upstate	Greenville, SC	Co-Investigator	Aqueous-PREP and PREPARE
William	Marshall			Prisma Health - Upstate	Greenville, SC	Co-Investigator	Aqueous-PREP and PREPARE
Shea B	Ray			Prisma Health - Upstate	Greenville, SC	Co-Investigator	Aqueous-PREP and PREPARE
Cory	Smith			Prisma Health - Upstate	Greenville, SC	Co-Investigator	Aqueous-PREP and PREPARE
Kyle M	Altman			Prisma Health - Upstate	Greenville, SC	Co-Investigator	Aqueous-PREP and PREPARE
Julia C	Quirion			Prisma Health - Upstate	Greenville, SC	Co-Investigator	Aqueous-PREP and PREPARE
Erin	Pichiotino			Prisma Health - Upstate	Greenville, SC	Co-Investigator	Aqueous-PREP and PREPARE
Markus F	Loeffler			Prisma Health - Upstate	Greenville, SC	Co-Investigator	Aqueous-PREP and PREPARE

*Indicates required information. Only first name, last name, and suffix will appear in PubMed.

*First Name and Middle Initial(s)	*Last Name	*Suffix (eg, Jr, III)	Academic Degrees	Institution	Location (city, state/province, country)	Role or Contribution, eg, chair, principal investigator	Group (if more than 1 Group listed in the byline) and/or Subgroup (eg, Steering Committee)
Austin A	Cole			Prisma Health - Upstate	Greenville, SC	Co-Investigator	Aqueous-PREP and PREPARE
Ethan J	Maltz			Prisma Health - Upstate	Greenville, SC	Co-Investigator	Aqueous-PREP and PREPARE
Wesley	Parker			Prisma Health - Upstate	Greenville, SC	Co-Investigator	Aqueous-PREP and PREPARE
T Bennett	Ramsey			Prisma Health - Upstate	Greenville, SC	Co-Investigator	Aqueous-PREP and PREPARE
Alex	Burnikel			Prisma Health - Upstate	Greenville, SC	Co-Investigator	Aqueous-PREP and PREPARE
Michael	Colello			Prisma Health - Upstate	Greenville, SC	Co-Investigator	Aqueous-PREP and PREPARE
Russell	Stewart			Prisma Health - Upstate	Greenville, SC	Co-Investigator	Aqueous-PREP and PREPARE
Jeremy	Wise			Prisma Health - Upstate	Greenville, SC	Co-Investigator	Aqueous-PREP and PREPARE
Matthew	Anderson			Prisma Health - Upstate	Greenville, SC	Co-Investigator	Aqueous-PREP and PREPARE
Joshua	Eskew			Prisma Health - Upstate	Greenville, SC	Co-Investigator	Aqueous-PREP and PREPARE
Benjamin	Judkins			Prisma Health - Upstate	Greenville, SC	Co-Investigator	Aqueous-PREP and PREPARE
James M	Miller			Prisma Health - Upstate	Greenville, SC	Co-Investigator	Aqueous-PREP and PREPARE
Stephanie L	Tanner			Prisma Health - Upstate	Greenville, SC	Research Coordinator	Aqueous-PREP and PREPARE
Rebecca G	Snider			Prisma Health - Upstate	Greenville, SC	Research Coordinator	Aqueous-PREP and PREPARE
Christine E	Townsend			Prisma Health - Upstate	Greenville, SC	Research Coordinator	Aqueous-PREP and PREPARE

*Indicates required information. Only first name, last name, and suffix will appear in PubMed.

***First Name and Middle Initial(s)**	***Last Name**	***Suffix (eg, Jr, III)**	Academic Degrees	Institution	Location (city, state/province, country)	Role or Contribution, eg, chair, principal investigator	Group (if more than 1 Group listed in the byline) and/or Subgroup (eg, Steering Committee)
Kayla H	Pham			Prisma Health - Upstate	Greenville, SC	Research Coordinator	Aqueous-PREP and PREPARE
Abigail	Martin			Prisma Health - Upstate	Greenville, SC	Research Coordinator	Aqueous-PREP and PREPARE
Emily	Robertson			Prisma Health - Upstate	Greenville, SC	Research Coordinator	Aqueous-PREP and PREPARE
Emily	Bray			Prisma Health - Upstate	Greenville, SC	Research Coordinator	Aqueous-PREP and PREPARE
J Wilson Sykes				Prisma Health - Upstate	Greenville, SC	Research Coordinator	Aqueous-PREP and PREPARE
Krystina	Yoder			Prisma Health - Upstate	Greenville, SC	Research Coordinator	Aqueous-PREP and PREPARE
Kelsey	Conner			Prisma Health - Upstate	Greenville, SC	Research Coordinator	Aqueous-PREP and PREPARE
Harper	Abbott			Prisma Health - Upstate	Greenville, SC	Research Coordinator	Aqueous-PREP and PREPARE
Saam	Morshed			University of California, San Francisco	San Francisco, CA	Local Principal Investigator	Aqueous-PREP and PREPARE
Meir	Mormor			University of California, San Francisco	San Francisco, CA	Local Principal Investigator	Aqueous-PREP and PREPARE
Theodore	Miclau			University of California, San Francisco	San Francisco, CA	Local Principal Investigator	Aqueous-PREP and PREPARE
Amir	Matityahu			University of California, San Francisco	San Francisco, CA	Co-Investigator	Aqueous-PREP and PREPARE
R Trigg	McClellan			University of California, San Francisco	San Francisco, CA	Co-Investigator	Aqueous-PREP and PREPARE
David	Shearer			University of California, San Francisco	San Francisco, CA	Co-Investigator	Aqueous-PREP and PREPARE
Paul	Toogood			University of California, San Francisco	San Francisco, CA	Co-Investigator	Aqueous-PREP and PREPARE

*Indicates required information. Only first name, last name, and suffix will appear in PubMed.

***First Name and Middle Initial(s)**	***Last Name**	***Suffix (eg, Jr, III)**	Academic Degrees	Institution	Location (city, state/province, country)	Role or Contribution, eg, chair, principal investigator	Group (if more than 1 Group listed in the byline) and/or Subgroup (eg, Steering Committee)
Anthony	Ding			University of California, San Francisco	San Francisco, CA	Co-Investigator	Aqueous-PREP and PREPARE
Jothi	Murali			University of California, San Francisco	San Francisco, CA	Co-Investigator	Aqueous-PREP and PREPARE
Ashraf	El Naga			University of California, San Francisco	San Francisco, CA	Co-Investigator	Aqueous-PREP and PREPARE
Jennifer	Tangtiphaiboontana			University of California, San Francisco	San Francisco, CA	Research Coordinator	Aqueous-PREP and PREPARE
Tigist	Belaye			University of California, San Francisco	San Francisco, CA	Research Coordinator	Aqueous-PREP and PREPARE
Eleni	Berhaneselase			University of California, San Francisco	San Francisco, CA	Research Coordinator	Aqueous-PREP and PREPARE
Dmitry	Pokhvashchey			University of California, San Francisco	San Francisco, CA	Research Coordinator	Aqueous-PREP and PREPARE
Joshua L	Gary			McGovern Medical School at UTHealth Houston	Houston, TX	Local Principal Investigator	Aqueous-PREP
Stephen J	Warner			McGovern Medical School at UTHealth Houston	Houston, TX	Co-Investigator	Aqueous-PREP
John W	Munz			McGovern Medical School at UTHealth Houston	Houston, TX	Co-Investigator	Aqueous-PREP
Andrew M	Choo			McGovern Medical School at UTHealth Houston	Houston, TX	Co-Investigator	Aqueous-PREP
Timothy S	Achor			McGovern Medical School at UTHealth Houston	Houston, TX	Co-Investigator	Aqueous-PREP
Milton L "Chip"	Routt			McGovern Medical School at UTHealth Houston	Houston, TX	Co-Investigator	Aqueous-PREP
Michael Kutzler	Kutzler			McGovern Medical School at UTHealth Houston	Houston, TX	Research Coordinator	Aqueous-PREP
Sterling	Boutte			McGovern Medical School at UTHealth Houston	Houston, TX	Research Coordinator	Aqueous-PREP

Supplemental Online Content: Nonauthor Collaborators

*Indicates required information. Only first name, last name, and suffix will appear in PubMed.

***First Name and Middle Initial(s)**	***Last Name**	***Suffix (eg, Jr, III)**	Academic Degrees	Institution	Location (city, state/province, country)	Role or Contribution, eg, chair, principal investigator	Group (if more than 1 Group listed in the byline) and/or Subgroup (eg, Steering Committee)
Ryan J	Warth			McGovern Medical School at UTHealth Houston	Houston, TX	Research Coordinator	Aqueous-PREP
Jennifer E	Hagen			University of Florida	Gainesville, FL	Local Principal Investigator	Aqueous-PREP
Matthew	Patrick			University of Florida	Gainesville, FL	Co-Investigator	Aqueous-PREP
Richard	Vlasak			University of Florida	Gainesville, FL	Co-Investigator	Aqueous-PREP
Thomas	Krupko			University of Florida	Gainesville, FL	Co-Investigator	Aqueous-PREP
Michael	Talerico			University of Florida	Gainesville, FL	Research Coordinator	Aqueous-PREP
Marybeth	Horodyski			University of Florida	Gainesville, FL	Research Coordinator	Aqueous-PREP
Marissa	Pazik			University of Florida	Gainesville, FL	Research Coordinator	Aqueous-PREP
Elizabeth	Lossada-Soto			University of Florida	Gainesville, FL	Research Coordinator	Aqueous-PREP
Niloofar	Dehghan			The CORE Institute	Phoenix, AZ	Local Principal Investigator	Aqueous-PREP
Michael	McKee			The CORE Institute	Phoenix, AZ	Co-Investigator	Aqueous-PREP
Clifford B	Jones			The CORE Institute	Phoenix, AZ	Local Principal Investigator	Aqueous-PREP
Debra L	Sietsema			The CORE Institute	Phoenix, AZ	Research Coordinator	Aqueous-PREP
Alyse	Williams			The CORE Institute	Phoenix, AZ	Research Coordinator	Aqueous-PREP
Tayler	Dykes			The CORE Institute	Phoenix, AZ	Research Coordinator	Aqueous-PREP
Ernesto	Guerra-Farfan			Vall d'Hebron University Hospital	Barcelona, Spain	Local Principal Investigator	Aqueous-PREP
Jordi	Thomas-Hernandez			Vall d'Hebron University Hospital	Barcelona, Spain		Aqueous-PREP
Jordi	Teixidor-Serra			Vall d'Hebron University Hospital	Barcelona, Spain		Aqueous-PREP

*Indicates required information. Only first name, last name, and suffix will appear in PubMed.

*First Name and Middle Initial(s)	*Last Name	*Suffix (eg, Jr, III)	Academic Degrees	Institution	Location (city, state/province, country)	Role or Contribution, eg, chair, principal investigator	Group (if more than 1 Group listed in the byline) and/or Subgroup (eg, Steering Committee)
Vicente	Molero-Garcia			Vall d'Hebron University Hospital	Barcelona, Spain		Aqueous-PREP
Jordi	Selga-Marsa			Vall d'Hebron University Hospital	Barcelona, Spain		Aqueous-PREP
Juan Antonio	Porcel-Vazquez			Vall d'Hebron University Hospital	Barcelona, Spain		Aqueous-PREP
Jose Vicente	Andres-Peiro			Vall d'Hebron University Hospital	Barcelona, Spain		Aqueous-PREP
Ignacio	Esteban-Feliu			Vall d'Hebron University Hospital	Barcelona, Spain		Aqueous-PREP
Nuria	Vidal-Tarrason			Vall d'Hebron University Hospital	Barcelona, Spain		Aqueous-PREP
Jordi	Serracanta			Vall d'Hebron University Hospital	Barcelona, Spain		Aqueous-PREP
Jorge	Nuñez-Camarena			Vall d'Hebron University Hospital	Barcelona, Spain		Aqueous-PREP
Maria	del Mar Villar-Casares			Vall d'Hebron University Hospital	Barcelona, Spain		Aqueous-PREP
Juame	Mestre-Torres			Vall d'Hebron University Hospital	Barcelona, Spain		Aqueous-PREP
Pilar	Lalueza-Broto			Vall d'Hebron University Hospital	Barcelona, Spain		Aqueous-PREP
Felipe	Moreira-Borim			Vall d'Hebron University Hospital	Barcelona, Spain		Aqueous-PREP
Yaiza	Garcia-Sanchez			Vall d'Hebron University Hospital	Barcelona, Spain	Research Coordinator	Aqueous-PREP
Francesc	Marcano-Fernández			Hospital Universitari Parc Tauli	Barcelona, Spain	Local Principal Investigator	Aqueous-PREP
Laia	Martínez-Carreres			Hospital Universitari Parc Tauli	Barcelona, Spain		Aqueous-PREP
David	Marti-Garin			Hospital Universitari Parc Tauli	Barcelona, Spain		Aqueous-PREP
Jorge	Serrano-Sanz			Hospital Universitari Parc Tauli	Barcelona, Spain		Aqueous-PREP
Joel	Sánchez-Fernández			Hospital Universitari Parc Tauli	Barcelona, Spain		Aqueous-PREP
Matsuyama	Sanz-Molero			Hospital Universitari Parc Tauli	Barcelona, Spain		Aqueous-PREP
Alejandro	Carballo			Hospital Universitari Parc Tauli	Barcelona, Spain		Aqueous-PREP
Xavier	Pelfort			Hospital Universitari Parc Tauli	Barcelona, Spain		Aqueous-PREP
Francesc	Acerboni-Flores			Hospital Universitari Parc Tauli	Barcelona, Spain		Aqueous-PREP
Anna	Alavedra-Massana			Hospital Universitari Parc Tauli	Barcelona, Spain		Aqueous-PREP

*Indicates required information. Only first name, last name, and suffix will appear in PubMed.

*First Name and Middle Initial(s)	*Last Name	*Suffix (eg, Jr, III)	Academic Degrees	Institution	Location (city, state/province, country)	Role or Contribution, eg, chair, principal investigator	Group (if more than 1 Group listed in the byline) and/or Subgroup (eg, Steering Committee)
Neus	Anglada-Torres			Hospital Universitari Parc Tauli	Barcelona, Spain		Aqueous-PREP
Alexandre	Berenguer			Hospital Universitari Parc Tauli	Barcelona, Spain		Aqueous-PREP
Jaume	Cámara-Cabrera			Hospital Universitari Parc Tauli	Barcelona, Spain		Aqueous-PREP
Ariadna	Caparros-García			Hospital Universitari Parc Tauli	Barcelona, Spain		Aqueous-PREP
Ferran	Fillat-Gomà			Hospital Universitari Parc Tauli	Barcelona, Spain		Aqueous-PREP
Ruben	Fuentes-López			Hospital Universitari Parc Tauli	Barcelona, Spain		Aqueous-PREP
Ramona	Garcia-Rodriguez			Hospital Universitari Parc Tauli	Barcelona, Spain		Aqueous-PREP
Nuria	Gimeno-Calavia			Hospital Universitari Parc Tauli	Barcelona, Spain		Aqueous-PREP
Marta	Martínez-Álvarez			Hospital Universitari Parc Tauli	Barcelona, Spain		Aqueous-PREP
Patricia	Martínez-Grau			Hospital Universitari Parc Tauli	Barcelona, Spain		Aqueous-PREP
Raúl	Pellejero-García			Hospital Universitari Parc Tauli	Barcelona, Spain		Aqueous-PREP
Ona	Ràfols-Perramon			Hospital Universitari Parc Tauli	Barcelona, Spain		Aqueous-PREP
Juan	Manuel Peñalver			Hospital Universitari Parc Tauli	Barcelona, Spain		Aqueous-PREP
Monica	Salomó Domènech			Hospital Universitari Parc Tauli	Barcelona, Spain		Aqueous-PREP
Albert	Soler-Cano			Hospital Universitari Parc Tauli	Barcelona, Spain		Aqueous-PREP
Aldo	Velasco-Barrera			Hospital Universitari Parc Tauli	Barcelona, Spain		Aqueous-PREP
Christian	Yela-Verdú			Hospital Universitari Parc Tauli	Barcelona, Spain		Aqueous-PREP
Mercedes	Bueno-Ruiz			Hospital Universitari Parc Tauli	Barcelona, Spain		Aqueous-PREP
Estrella	Sánchez-Palomino			Hospital Universitari Parc Tauli	Barcelona, Spain		Aqueous-PREP
Vito	Andriola			Hospital Universitari Parc Tauli	Barcelona, Spain		Aqueous-PREP

*Indicates required information. Only first name, last name, and suffix will appear in PubMed.

*First Name and Middle Initial(s)	*Last Name	*Suffix (eg, Jr, III)	Academic Degrees	Institution	Location (city, state/province, country)	Role or Contribution, eg, chair, principal investigator	Group (if more than 1 Group listed in the byline) and/or Subgroup (eg, Steering Committee)
Matilde	Molina-Corbacho			Hospital Universitari Parc Tauli	Barcelona, Spain		Aqueous-PREP
Yeray	Maldonado-Sotoca			Hospital Universitari Parc Tauli	Barcelona, Spain		Aqueous-PREP
Alfons	Gasset-Teixidor			Hospital Universitari Parc Tauli	Barcelona, Spain		Aqueous-PREP
Jorge	Blasco-Moreu			Hospital Universitari Parc Tauli	Barcelona, Spain		Aqueous-PREP
Núria	Fernández-Poch			Hospital Universitari Parc Tauli	Barcelona, Spain		Aqueous-PREP
Josep	Rodoreda-Puigdemasa			Hospital Universitari Parc Tauli	Barcelona, Spain		Aqueous-PREP
Arnau	Verdaguer-Figuerola			Hospital Universitari Parc Tauli	Barcelona, Spain		Aqueous-PREP
Heber Enrique	Cueva-Sevieri			Hospital Universitari Parc Tauli	Barcelona, Spain		Aqueous-PREP
Santiago	Garcia-Gimenez			Hospital Universitari Parc Tauli	Barcelona, Spain		Aqueous-PREP
William T	Obremsky			Vanderbilt Medical Center	Nashville, TN	Local Principal Investigator	Aqueous-PREP
Amir Alex	Jahangir			Vanderbilt Medical Center	Nashville, TN	Co-Investigator	Aqueous-PREP
Manish	Sethi			Vanderbilt Medical Center	Nashville, TN	Co-Investigator	Aqueous-PREP
Robert	Boyce			Vanderbilt Medical Center	Nashville, TN	Co-Investigator	Aqueous-PREP
Daniel J	Stinner			Vanderbilt Medical Center	Nashville, TN	Co-Investigator	Aqueous-PREP
Phillip	Mitchell			Vanderbilt Medical Center	Nashville, TN	Co-Investigator	Aqueous-PREP
Karen	Trochez			Vanderbilt Medical Center	Nashville, TN	Research Coordinator	Aqueous-PREP
Elsa	Rodriguez			Vanderbilt Medical Center	Nashville, TN	Research Coordinator	Aqueous-PREP
Charles	Pritchett			Vanderbilt Medical Center	Nashville, TN	Research Coordinator	Aqueous-PREP
Natalie	Hogan			Vanderbilt Medical Center	Nashville, TN	Research Coordinator	Aqueous-PREP

Supplemental Online Content: Nonauthor Collaborators

*Indicates required information. Only first name, last name, and suffix will appear in PubMed.

***First Name and Middle Initial(s)**	***Last Name**	***Suffix (eg, Jr, III)**	Academic Degrees	Institution	Location (city, state/province, country)	Role or Contribution, eg, chair, principal investigator	Group (if more than 1 Group listed in the byline) and/or Subgroup (eg, Steering Committee)
A Fidel	Moreno			Vanderbilt Medical Center	Nashville, TN	Research Coordinator	Aqueous-PREP
Christina	Boulton			Banner University Medical Center – Tucson	Tucson, AZ	Local Principal Investigator	Aqueous-PREP
Jason	Lowe			Banner University Medical Center – Tucson	Tucson, AZ	Co-Investigator	Aqueous-PREP
John T	Ruth			Banner University Medical Center – Tucson	Tucson, AZ	Co-Investigator	Aqueous-PREP
Brad	Askam			Banner University Medical Center – Tucson	Tucson, AZ	Co-Investigator	Aqueous-PREP
Andrea	Seach			Banner University Medical Center – Tucson	Tucson, AZ	Manager	Aqueous-PREP
Alejandro	Cruz			Banner University Medical Center – Tucson	Tucson, AZ	Research Coordinator	Aqueous-PREP
Breanna	Featherston			Banner University Medical Center – Tucson	Tucson, AZ	Research Coordinator	Aqueous-PREP
Robin	Carlson			Banner University Medical Center – Tucson	Tucson, AZ	Research Coordinator	Aqueous-PREP
Iliana	Romero			Banner University Medical Center – Tucson	Tucson, AZ	Research Coordinator	Aqueous-PREP
Isaac	Zarif			Banner University Medical Center – Tucson	Tucson, AZ	Research Coordinator	Aqueous-PREP
Michael	Prayson			Wright State University	Dayton, OH	Local Principal Investigator	Aqueous-PREP
Indresh	Venkatarayappa			Wright State University	Dayton, OH	Co-Investigator	Aqueous-PREP
Brandon	Horne			Wright State University	Dayton, OH	Co-Investigator	Aqueous-PREP
Jennifer	Jerele			Wright State University	Dayton, OH	Co-Investigator	Aqueous-PREP
Linda	Clark			Wright State University	Dayton, OH	Research Coordinator	Aqueous-PREP

Supplemental Online Content: Nonauthor Collaborators

*Indicates required information. Only first name, last name, and suffix will appear in PubMed.

*First Name and Middle Initial(s)	*Last Name	*Suffix (eg, Jr, III)	Academic Degrees	Institution	Location (city, state/province, country)	Role or Contribution, eg, chair, principal investigator	Group (if more than 1 Group listed in the byline) and/or Subgroup (eg, Steering Committee)
Nicholas M	Romeo			MetroHealth Medical Center	Cleveland, OH	Local Principal Investigator	PREPARE
Heather A	Vallier			MetroHealth Medical Center	Cleveland, OH	Co-Investigator	PREPARE
Anna	Vergon			MetroHealth Medical Center	Cleveland, OH	Research Assistant	PREPARE
Darius G	Viskontas			FRASER HEALTH AUTHORITY/Royal Columbian Hospital	New Westminster, BC	Local Principal Investigator	PREPARE
Kelly L	Apostle			FRASER HEALTH AUTHORITY/Royal Columbian Hospital	New Westminster, BC	Co-Investigator	PREPARE
Dory S	Boyer			FRASER HEALTH AUTHORITY/Royal Columbian Hospital	New Westminster, BC	Co-Investigator	PREPARE
Farhad O	Moola			FRASER HEALTH AUTHORITY/Royal Columbian Hospital	New Westminster, BC	Co-Investigator	PREPARE
Bertrand H	Perey			FRASER HEALTH AUTHORITY/Royal Columbian Hospital	New Westminster, BC	Co-Investigator	PREPARE
Trevor B	Stone			FRASER HEALTH AUTHORITY/Royal Columbian Hospital	New Westminster, BC	Co-Investigator	PREPARE
H Michael	Lemke			FRASER HEALTH AUTHORITY/Royal Columbian Hospital	New Westminster, BC	Co-Investigator	PREPARE
Ella	Spicer			FRASER HEALTH AUTHORITY/Royal Columbian Hospital	New Westminster, BC	Research Coordinator	PREPARE
Krysten	Payne			FRASER HEALTH AUTHORITY/Royal Columbian Hospital	New Westminster, BC	Research Assistant	PREPARE

Supplemental Online Content: Nonauthor Collaborators

*Indicates required information. Only first name, last name, and suffix will appear in PubMed.

***First Name and Middle Initial(s)**	***Last Name**	***Suffix (eg, Jr, III)**	Academic Degrees	Institution	Location (city, state/province, country)	Role or Contribution, eg, chair, principal investigator	Group (if more than 1 Group listed in the byline) and/or Subgroup (eg, Steering Committee)
Kevin	Phelps			Carolinas Medical Center, Atrium Health Musculoskeletal Institute	Charlotte, NC	Local Principal Investigator	PREPARE
Michael	Bosse			Carolinas Medical Center, Atrium Health Musculoskeletal Institute	Charlotte, NC	Co-Investigator	PREPARE
Madhav	Karunakar			Carolinas Medical Center, Atrium Health Musculoskeletal Institute	Charlotte, NC	Co-Investigator	PREPARE
Laurence	Kempton			Carolinas Medical Center, Atrium Health Musculoskeletal Institute	Charlotte, NC	Co-Investigator	PREPARE
Stephen	Sims			Carolinas Medical Center, Atrium Health Musculoskeletal Institute	Charlotte, NC	Co-Investigator	PREPARE
Joseph	Hsu			Carolinas Medical Center, Atrium Health Musculoskeletal Institute	Charlotte, NC	Co-Investigator	PREPARE
Rachel	Seymour			Carolinas Medical Center, Atrium Health Musculoskeletal Institute	Charlotte, NC	Manager	PREPARE
Christine	Churchill			Carolinas Medical Center, Atrium Health Musculoskeletal Institute	Charlotte, NC	Research Coordinator	PREPARE
Ada	Mayfield			Carolinas Medical Center, Atrium Health Musculoskeletal Institute	Charlotte, NC	Research Assistant	PREPARE
Juliette	Sweeney			Carolinas Medical Center, Atrium Health Musculoskeletal Institute	Charlotte, NC	Research Assistant	PREPARE
Robert A	Hymes			Inova Fairfax Medical Campus	Falls Church, VA	Local Principal Investigator	PREPARE
Cary C	Schwartzbach			Inova Fairfax Medical Campus	Falls Church, VA	Co-Investigator	PREPARE
Jeff E	Schulman			Inova Fairfax Medical Campus	Falls Church, VA	Co-Investigator	PREPARE
A Stephen	Malekzadeh			Inova Fairfax Medical Campus	Falls Church, VA	Co-Investigator	PREPARE
Michael A	Holzman			Inova Fairfax Medical Campus	Falls Church, VA	Co-Investigator	PREPARE
Greg E	Gaski			Inova Fairfax Medical Campus	Falls Church, VA	Co-Investigator	PREPARE
Johnathan	Wills			Inova Fairfax Medical Campus	Falls Church, VA	Research Coordinator	PREPARE
Holly	Pilson			Wake Forest Baptist Health	Winston-Salem, NC	Local Principal Investigator	PREPARE

Supplemental Online Content: Nonauthor Collaborators

*Indicates required information. Only first name, last name, and suffix will appear in PubMed.

***First Name and Middle Initial(s)**	***Last Name**	***Suffix (eg, Jr, III)**	Academic Degrees	Institution	Location (city, state/province, country)	Role or Contribution, eg, chair, principal investigator	Group (if more than 1 Group listed in the byline) and/or Subgroup (eg, Steering Committee)
Eben A	Carroll			Wake Forest Baptist Health	Winston-Salem, NC	Co-Investigator	PREPARE
Jason J	Halvorson			Wake Forest Baptist Health	Winston-Salem, NC	Co-Investigator	PREPARE
Sharon	Babcock			Wake Forest Baptist Health	Winston-Salem, NC	Co-Investigator	PREPARE
J Brett	Goodman			Wake Forest Baptist Health	Winston-Salem, NC	Research Coordinator	PREPARE
Martha B	Holden			Wake Forest Baptist Health	Winston-Salem, NC	Research Assistant	PREPARE
Wendy	Williams			Wake Forest Baptist Health	Winston-Salem, NC	Research Assistant	PREPARE
Taylor	Hill			Wake Forest Baptist Health	Winston-Salem, NC	Research Assistant	PREPARE
Ariel	Brotherton			Wake Forest Baptist Health	Winston-Salem, NC	Research Assistant	PREPARE
Thomas F	Higgins			University of Utah	Salt Lake City, Utah	Local Principal Investigator	PREPARE
Justin M	Haller			University of Utah	Salt Lake City, Utah	Co-Investigator	PREPARE
David L	Rothberg			University of Utah	Salt Lake City, Utah	Co-Investigator	PREPARE
Lucas S	Marchand			University of Utah	Salt Lake City, Utah	Co-Investigator	PREPARE
Zachary M	Olsen			University of Utah	Salt Lake City, Utah	Co-Investigator	PREPARE
Abby V	McGowan			University of Utah	Salt Lake City, Utah	Research Coordinator	PREPARE
Sophia	Hill			University of Utah	Salt Lake City, Utah	Research Coordinator	PREPARE
Morgan K	Dauk			University of Utah	Salt Lake City, Utah	Research Coordinator	PREPARE
I Leah	Gitajn			Dartmouth-Hitchcock Medical Center	Lebanon, NH	Local Principal Investigator	PREPARE
Marcus	Coe			Dartmouth-Hitchcock Medical Center	Lebanon, NH	Co-Investigator	PREPARE
Kevin	Dwyer			Dartmouth-Hitchcock Medical Center	Lebanon, NH	Co-Investigator	PREPARE
Devin S	Mullin			Dartmouth-Hitchcock Medical Center	Lebanon, NH	Research Coordinator	PREPARE
Theresa A	Chockbengboun			Dartmouth-Hitchcock Medical Center	Lebanon, NH	Research Assistant	PREPARE

Supplemental Online Content: Nonauthor Collaborators

*Indicates required information. Only first name, last name, and suffix will appear in PubMed.

*First Name and Middle Initial(s)	*Last Name	*Suffix (eg, Jr, III)	Academic Degrees	Institution	Location (city, state/province, country)	Role or Contribution, eg, chair, principal investigator	Group (if more than 1 Group listed in the byline) and/or Subgroup (eg, Steering Committee)
Peter A	DePalo	Sr		Dartmouth-Hitchcock Medical Center	Lebanon, NH	Research Assistant	PREPARE
Marilyn	Heng			Massachusetts General Hospital	Boston, MA	Local Principal Investigator	PREPARE
Mitchel B	Harris			Massachusetts General Hospital	Boston, MA	Co-Investigator	PREPARE
David W	Lhowe			Massachusetts General Hospital	Boston, MA	Co-Investigator	PREPARE
John G	Esposito			Massachusetts General Hospital	Boston, MA	Co-Investigator	PREPARE
Ahmad	Alnasser			Massachusetts General Hospital	Boston, MA	Research Assistant	PREPARE
Steven F	Shannon			Bryan Medical Center	Lincoln, NB	Co-Investigator	PREPARE
Alesha N	Scott			Bryan Medical Center	Lincoln, NB	Co-Investigator	PREPARE
Bobbi	Clinch			Bryan Medical Center	Lincoln, NB	Research Coordinator	PREPARE
Becky	Webber			Bryan Medical Center	Lincoln, NB	Research Coordinator	PREPARE
Michael J	Beltran			University of Cincinnati	Cincinnati, OH	Co-Investigator	PREPARE
Michael T	Archdeacon			University of Cincinnati	Cincinnati, OH	Co-Investigator	PREPARE
Henry Claude	Sagi			University of Cincinnati	Cincinnati, OH	Co-Investigator	PREPARE
John D	Wyrick			University of Cincinnati	Cincinnati, OH	Co-Investigator	PREPARE
Theodore Toan	Le			University of Cincinnati	Cincinnati, OH	Co-Investigator	PREPARE
Richard T	Laughlin			University of Cincinnati	Cincinnati, OH	Co-Investigator	PREPARE
Cameron G	Thomson			University of Cincinnati	Cincinnati, OH	Research Coordinator	PREPARE
Kimberly	Hasselfeld			University of Cincinnati	Cincinnati, OH	Research Coordinator	PREPARE
Carol A	Lin			Cedars-Sinai Medical Center	Los Angeles, CA	Co-Investigator	PREPARE
Mark S	Vrahas			Cedars-Sinai Medical Center	Los Angeles, CA	Co-Investigator	PREPARE
Charles N	Moon			Cedars-Sinai Medical Center	Los Angeles, CA	Co-Investigator	PREPARE
Milton T	Little			Cedars-Sinai Medical Center	Los Angeles, CA	Co-Investigator	PREPARE
Geoffrey S	Marecek			Cedars-Sinai Medical Center	Los Angeles, CA	Co-Investigator	PREPARE
Denice M	Dubaclet			Cedars-Sinai Medical Center	Los Angeles, CA	Research Coordinator	PREPARE

*Indicates required information. Only first name, last name, and suffix will appear in PubMed.

***First Name and Middle Initial(s)**	***Last Name**	***Suffix (eg, Jr, III)**	Academic Degrees	Institution	Location (city, state/province, country)	Role or Contribution, eg, chair, principal investigator	Group (if more than 1 Group listed in the byline) and/or Subgroup (eg, Steering Committee)
John A	Scolaro			University of California, Irvine	Irvine, CA	Co-Investigator	PREPARE
James R	Learned			University of California, Irvine	Irvine, CA	Co-Investigator	PREPARE
Philip K	Lim			University of California, Irvine	Irvine, CA	Co-Investigator	PREPARE
Susan	Demas			University of California, Irvine	Irvine, CA	Co-Investigator	PREPARE
Arya	Amirhekmat			University of California, Irvine	Irvine, CA	Research Coordinator	PREPARE
Yan Marco	Dela Cruz			University of California, Irvine	Irvine, CA	Research Coordinator	PREPARE
Patrick F	Bergin			University of Mississippi Medical Center	Jackson, MS	Local Principal Investigator	PREPARE
George V	Russell			University of Mississippi Medical Center	Jackson, MS	Co-Investigator	PREPARE
Matthew L	Graves			University of Mississippi Medical Center	Jackson, MS	Co-Investigator	PREPARE
John	Morellato			University of Mississippi Medical Center	Jackson, MS	Co-Investigator	PREPARE
Sheketha L	McGee			University of Mississippi Medical Center	Jackson, MS	Research Coordinator	PREPARE
Eldrin L	Bhanat			University of Mississippi Medical Center	Jackson, MS	Research Assistant	PREPARE
Ugur	Yener			University of Mississippi Medical Center	Jackson, MS	Research Coordinator	PREPARE
Rajinder	Khanna			University of Mississippi Medical Center	Jackson, MS	Research Coordinator	PREPARE
Priyanka	Nehete			University of Mississippi Medical Center	Jackson, MS	Research Coordinator	PREPARE
Samir	Mehta			University of Pennsylvania	Philadelphia, PA	Local Principal Investigator	PREPARE
Derek	Donehan			University of Pennsylvania	Philadelphia, PA	Co-Investigator	PREPARE
Annamarie	Horan			University of Pennsylvania	Philadelphia, PA	Manager	PREPARE
Mary	Dooley			University of Pennsylvania	Philadelphia, PA	Research Assistant	PREPARE

Supplemental Online Content: Nonauthor Collaborators

*Indicates required information. Only first name, last name, and suffix will appear in PubMed.

***First Name and Middle Initial(s)**	***Last Name**	***Suffix (eg, Jr, III)**	Academic Degrees	Institution	Location (city, state/province, country)	Role or Contribution, eg, chair, principal investigator	Group (if more than 1 Group listed in the byline) and/or Subgroup (eg, Steering Committee)
David	Potter			Sanford Health	Sioux Falls, SD	Local Principal Investigator	PREPARE
Robert	VanDemark	III		Sanford Health	Sioux Falls, SD	Co-Investigator	PREPARE
Kyle	Seabold			Sanford Health	Sioux Falls, SD	Research Coordinator	PREPARE
Nicholas	Staudenmier			Sanford Health	Sioux Falls, SD	Research Coordinator	PREPARE
Michael J	Weaver			Brigham Women's Hospital	Boston, MA	Local Principal Investigator	PREPARE
Arvind G	von Keudell			Brigham Women's Hospital	Boston, MA	Co-Investigator	PREPARE
Abigail E	Sagona			Brigham Women's Hospital	Boston, MA	Research Assistant	PREPARE
Todd	Jaeblon			University of Maryland Prince George's Capital Region Health	Cheverly, MD	Local Principal Investigator	PREPARE
Robert	Beer			University of Maryland Prince George's Capital Region Health	Cheverly, MD	Co-Investigator	PREPARE
Haley K	Demyanovich			University of Maryland Prince George's Capital Region Health	Cheverly, MD	Research Coordinator	PREPARE
Brent	Bauer			University of Maryland Prince George's Capital Region Health	Cheverly, MD	Research Coordinator	PREPARE
Sean	Meredith			University of Maryland Prince George's Capital Region Health	Cheverly, MD	Research Coordinator	PREPARE
Sneh	Talwar			University of Maryland Prince George's Capital Region Health	Cheverly, MD	Research Coordinator	PREPARE
Christopher M	Domes			University of Wisconsin Madison	Madison, WI	Local Principal Investigator	PREPARE
Mark J	Gage			Duke University Hospital	Durham, NC	Local Principal Investigator	PREPARE
Rachel M	Reilly			Duke University Hospital	Durham, NC	Co-Investigator	PREPARE
Ariana	Paniagua			Duke University Hospital	Durham, NC	Research Coordinator	PREPARE

Supplemental Online Content: Nonauthor Collaborators

*Indicates required information. Only first name, last name, and suffix will appear in PubMed.

***First Name and Middle Initial(s)**	***Last Name**	***Suffix (eg, Jr, III)**	Academic Degrees	Institution	Location (city, state/province, country)	Role or Contribution, eg, chair, principal investigator	Group (if more than 1 Group listed in the byline) and/or Subgroup (eg, Steering Committee)
JaNell	Depree			Duke University Hospital	Durham, NC	Research Coordinator	PREPARE
