# Supplementary material for: Association of Patient-Level and Hospital-Level Factors With Timely Fracture Care by Race
Source: JAMA Netw Open. 2022 Nov 30;5(11):e2244357. doi: 10.1001/jamanetworkopen.2022.44357 (PMC9713603; doi:10.1001/jamanetworkopen.2022.44357)
Supplement: Supplement 3. — Data Sharing Statement [file jamanetwopen-e2244357-s003.pdf]

Gitajn IL, Werth P, Fernandes E, et al; on behalf of PREP-IT Investigators. Association of Patient-Level and Hospital-Level Factors With Timely Fracture Care by Race. *JAMA Netw Open.* 2022;5(11):e2244357. doi:10.1001/jamanetworkopen.2022.44357

## Data Sharing Statement

### Data

**Data available:** No

### Additional Information

**Explanation for why data not available:** The data collected for this study is not open source, however, if there are questions/issues the data may be shared by study investigators.
